# Rare Malignant Transformation of a Granular Cell Tumor: A Case of Recurrent Perianal Lesion With Pulmonary and Lymph Node Metastases

**DOI:** 10.7759/cureus.86908

**Published:** 2025-06-28

**Authors:** Kradi Yassin, Soufiane Taibi, Mouad Ouryemchi, Abdelali Guellil, Mohammed Bouziane

**Affiliations:** 1 Department of General Surgery, Faculty of Medicine and Pharmacy, Laboratory of Anatomy, Microsurgery and Surgery Experimental and Medical Simulation (LAMCESM), Mohammed 1st University, Oujda, MAR; 2 Department of Visceral Surgery and Digestive Oncology, Mohammed VI University Hospital, Oujda, MAR; 3 Department of General Surgery, Centre Hospitalier Universitaire Mohammed VI de Marrakech, Oujda, MAR

**Keywords:** abrikossoff tumor, granular cell tumor, lymph node metastases, neurogenic schwannian neoplasms, pulmonary metastases

## Abstract

This report presents the case of a 52-year-old woman with a recurrent perianal mass, initially diagnosed as a benign GCT. Despite surgical resection, the tumor later metastasized to the lungs and lymph nodes, indicating malignant transformation. Imaging and histological analysis confirmed the malignancy, with cellular atypia and metastasis. The patient declined further treatment and passed away shortly thereafter. This case underscores the potential for malignant transformation in GCTs, particularly in recurrent cases. While surgical removal with clear margins remains the standard approach, malignant GCTs often present a poor prognosis due to high rates of recurrence and metastasis, with limited response to chemotherapy and radiation therapy.

## Introduction

Granular cell tumors (GCTs) are neurogenic schwannian neoplasms [1], a rare entity representing less than 5% of soft tissue tumors [2], first described by Abrikossoff in 1926 [3]. These tumors are generally benign, with only 0.5% to 2% of cases reported as malignant [4]. They primarily affect the oral mucosa and the subcutaneous tissue of the head and neck.

The malignant nature of GCTs may be suggested by cellular polymorphism, mitotic activity, rapid recurrence, and especially the occurrence of metastases. Surgical resection with clear margins is the cornerstone of GCT management.

Magnetic resonance imaging (MRI) remains the best radiological tool for characterizing these tumors. The diagnosis is based especially on histological examination, while tumor cell immunostaining confirms the schwannian nature of the tumor.

We report the case of pulmonary and lymph node metastases complicating a recurrent perianal Abrikossoff tumor, highlighting the potential for late transformation of these tumors, which are typically benign and have a favorable prognosis.

## Case presentation

We report the case of a 52-year-old patient, with no significant medical history, who presented with a right perianal mass associated with anal pain exacerbated by defecation, evolving for eight months prior to her admission. Clinical examination at admission revealed a 6-cm right perianal mass, fixed to the underlying tissues and painful. No abnormalities were found on digital rectal examination. There were no other notable findings, including no weight loss or general health deterioration.

**Figure 1:**
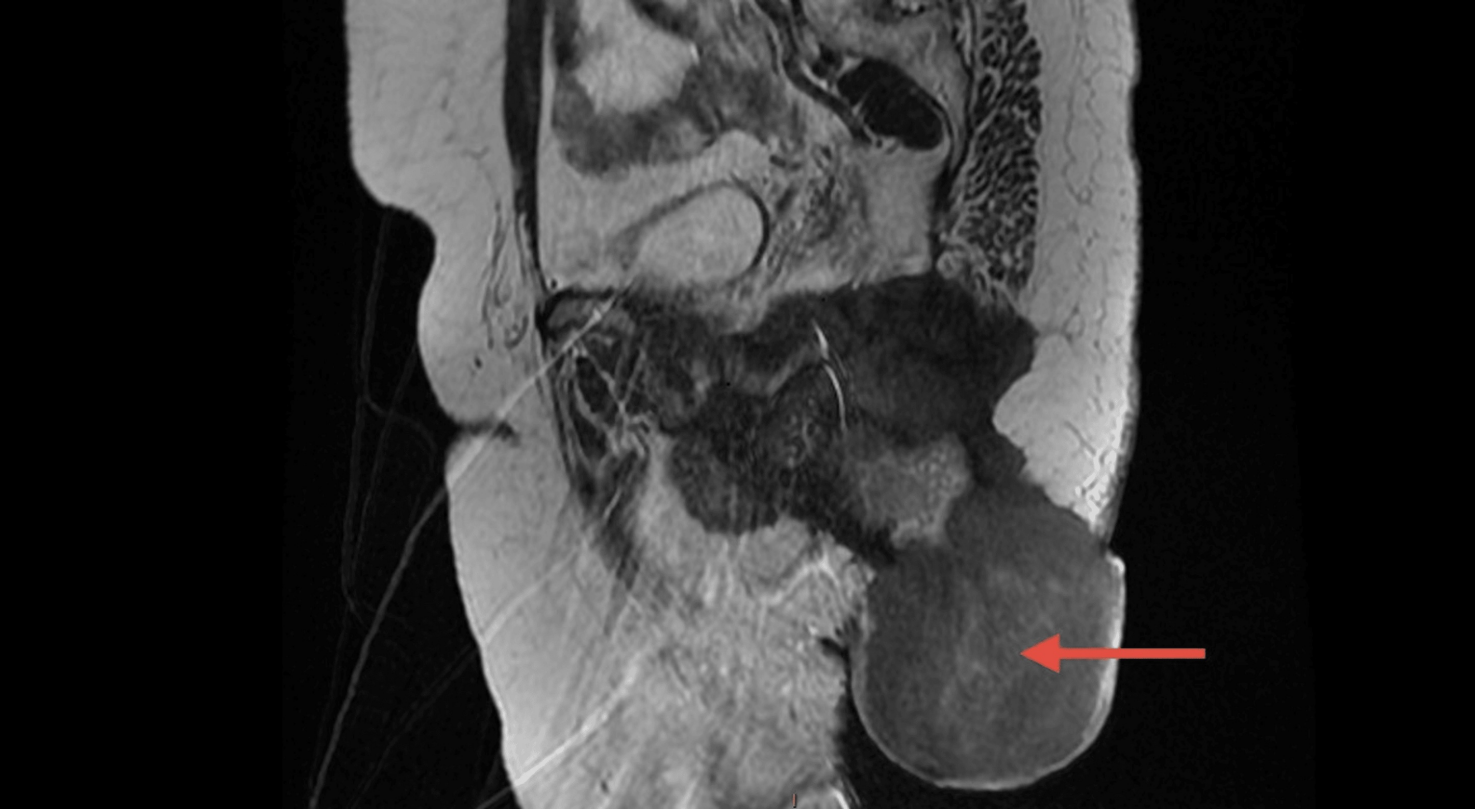
Sagittal section of a pelvic MRI Arrow points to the mass infiltrating the ischiopubic fossa with irregular contours, poorly defined, invading the levator ani muscle, measuring 70x62 mm.

Blood tests, including tumor markers (carbohydrate antigen 19-9 and carcinoembryonic antigen), were normal. A pelvic MRI was performed, which revealed a mass infiltrating the right ischioanal fossa, measuring 70x62 mm, with irregular borders, poorly defined on T1 and hypointense on T2 images. The mass showed progressive and delayed enhancement after gadolinium injection, infiltrating the levator ani muscles, with its walls being thin and irregular. It was in close contact with the gluteus maximus muscle without any clear separation (Figure 1).

A low-endoscopy was also performed, and progression up to 50 cm from the anal margin showed no abnormalities. Initially, a surgical biopsy was performed, and histological examination revealed fibro-adipose tissue with a mononuclear inflammatory infiltrate composed of lymphocytes and plasma cells, with no evidence of tumor proliferation. The case was discussed in the multidisciplinary meeting, and the decision to perform surgical resection with clear margins was made, for which the peripheral fibers of the anal sphincter had to be resected and the sphincter had to be repaired. Post-surgical follow-up was uneventful without any defecation disorder, and the patient was discharged three days after surgery.

**Figure 2:**
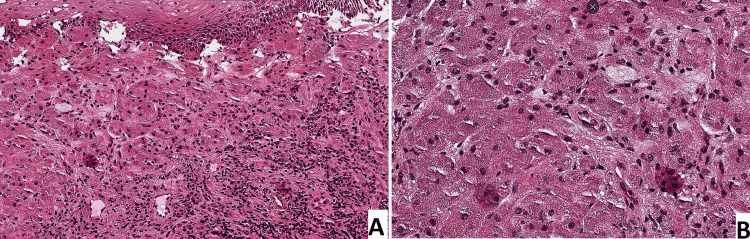
A. Microscopic image showing a proliferation of sheets and ribbons of cells separated by fine bands of collagen (HE, x200). B. Higher magnification microscopic image showing polygonal cells with small, dense nuclei. The cytoplasm is abundant and eosinophilic with coarse granules (HE, x400).

Histological study showed a fibrous and skeletal striated muscle tissue, extensively infiltrated by tumor proliferation in trabecular and nest-like formations. The tumor cells were polygonal, large in size, with abundant eosinophilic and granular cytoplasm, and a regular, slightly hyperchromatic nucleus with no mitosis (Figure 2). Immunohistochemistry showed that the tumor cells expressed PS100. The diagnosis of GCT was made with no signs of malignancy. Given these results, the decision of wait-and-see protocol was made, and a CT scan was scheduled every six months.

**Figure 3:**
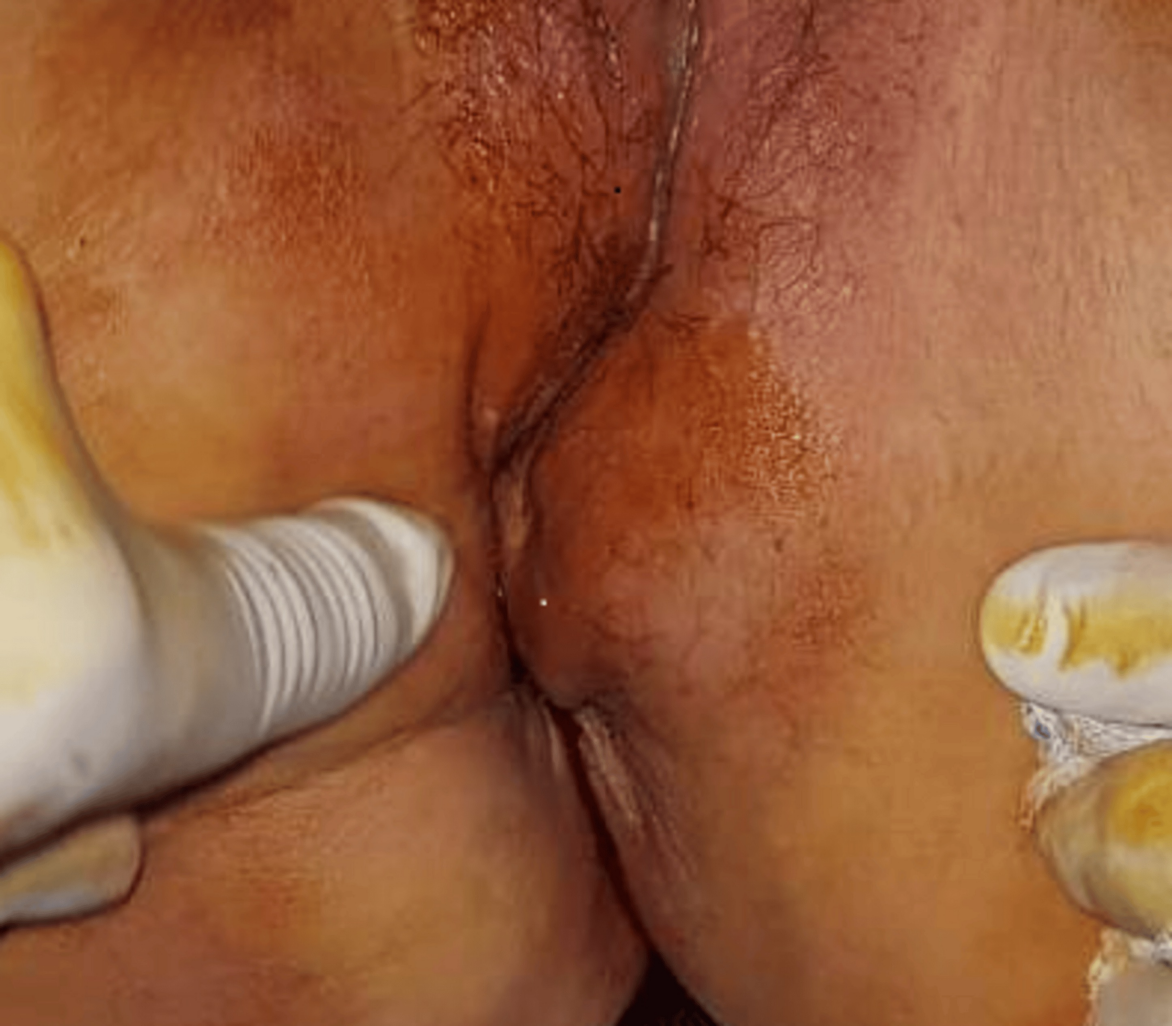
Frontal view showing swelling of the left para-anal region pushing the anus and narrowing the anal canal lumen.

Several months later, the evolution was marked by the appearance of proctalgia with a deterioration of general health. Physical examination revealed two masses: a hard-right perianal mass displacing the right rectal wall and protruding into the vagina, and a second left perianal mass displacing the wall and narrowing the anal canal (Figure 3).

**Figure 4:**
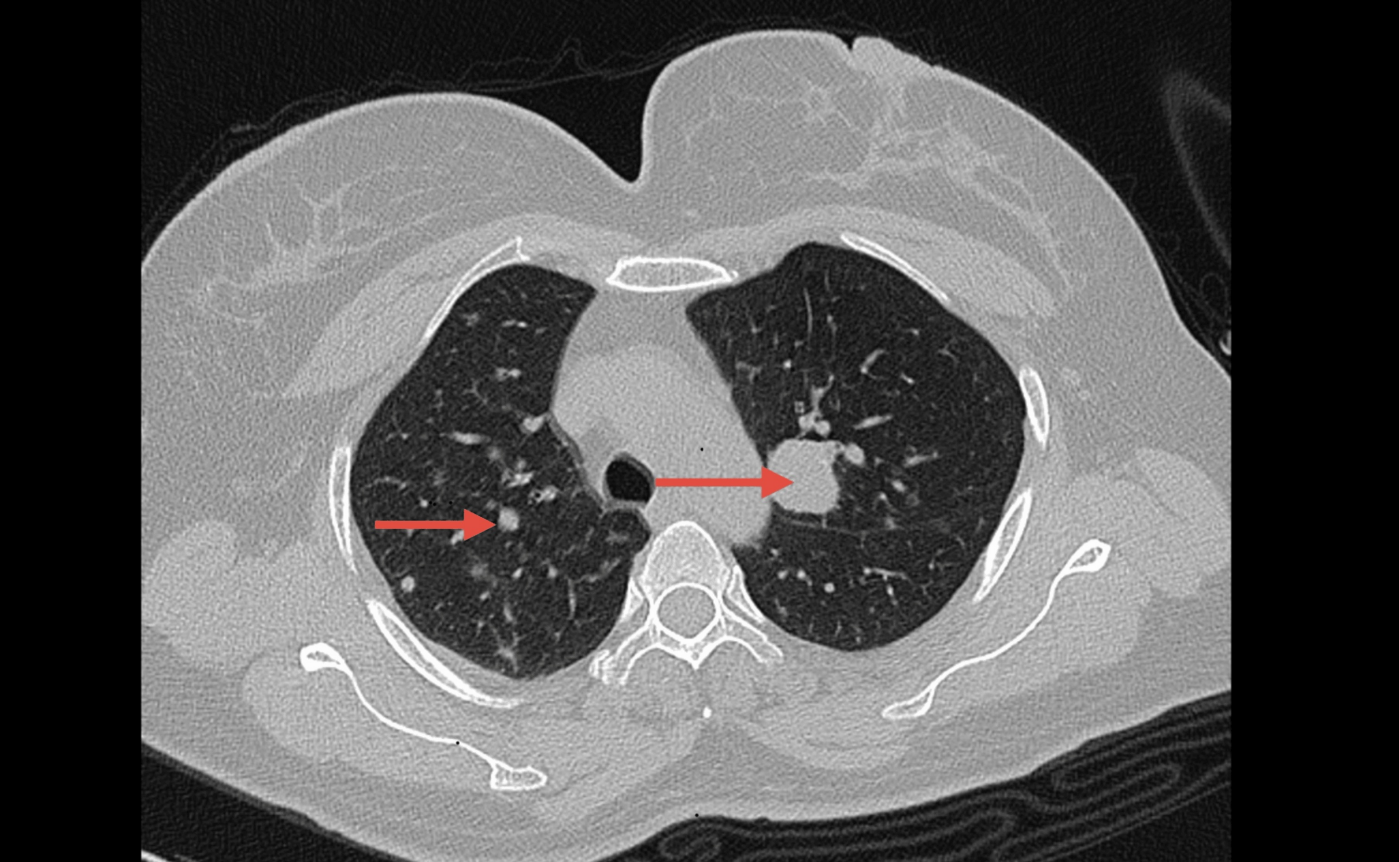
Cross-sectional image of a chest CT scan Arrow indicates multiple round opacities disseminated in both lung fields.

In light of the tumor recurrence, a thoraco-abdomino-pelvic CT scan was first performed, which revealed a poorly defined tissue mass centered on the right ischioanal region with irregular contours, enhancing after contrast injection, measuring 80x81 mm, reaching the right ischiopubic branch and the external obturator muscle. The mass infiltrated the right gluteus muscles posteriorly, the lower third of the vagina, and the anal canal. Additionally, the CT scan showed several bilateral pulmonary parenchymal nodules and micronodules, with the largest measuring 13x10 mm in the left upper lobe and 6x6 mm in the left lower lobe (Figure 4).

Against these findings, we suspected a malignant transformation. A surgical biopsy of the right perianal mass and trucut biopsy of the left perianal mass were performed, and histological and immunohistochemical examination confirmed the presence of a malignant GCT. The patient was referred to oncology department, but she refused any further investigations or treatment and died a few weeks later.

## Discussion

The Abrikossoff tumor, or GCT, is a rare neoplasm that accounts for less than 5% of all soft tissue tumors [2]. It was first described in 1926 by Russian pathologist Alexei Ivanovich Abrikossoff, who initially referred to it as "myoblastomas," due to the proposed skeletal muscle origin [3]. It was not until 1949 that Frest and Custer demonstrated the presence of myelin degradation products, suggesting a neural origin. Currently, the schwannian neurogenic origin is accepted, as confirmed by immunohistochemistry [5].

GCTs can occur at any age, with a peak incidence between the fourth and sixth decades of life [6]. They affect women in two-thirds of cases [7], with a particular predominance among the African-American population. According to studies, multifocal presentations occur in only 5-7% of cases [8]. These tumors can arise anywhere in the body, but gastrointestinal tract involvement accounts for only 8% of all GCTs [9], with the esophagus being the most common site. Moreover, perianal involvement is extremely rare.

MRI remains the best radiological tool for characterizing these tumors. On T1-weighted images, the lesions appear round or oval, isointense with muscle. On T2-weighted images, the lesions are typically iso-intense with muscle, with peripheral enhancement. In malignant forms, invasion of adjacent structures can be observed [10].

The diagnosis is based especially on histological examination, where these neoplasms are characterized by large tumor cells with abundant eosinophilic and granular cytoplasm and round or oval nuclei. These polygonal cells are grouped in clusters and delineated by fibrous stroma. Tumor cell immunostaining for S100 protein, neuron-specific enolase, and CD68 confirms the schwannian nature of the tumor [11]. Vascular and/or perineural invasion may be observed but does not constitute a definitive sign of malignancy.

GCTs are malignant in 0.5-2% of cases [4-12], with a very high potential for local recurrence and metastasis. The most common sites of metastatic spread are regional lymph nodes, the lungs, the liver, and bones. The malignant potential of Abrikossoff tumor can be indicated by clinical or histological criteria, and the occurrence of lymph node or systemic metastases remains the most important factor in defining malignancy, although not all malignant GCTs present with metastases [13]. Clinically, factors such as female sex, older age, large tumor size (>5 cm), invasion of adjacent structures, and rapid growth are characteristics that can predict malignancy.

From a histological perspective, Fanburg-Smith et al. reported that the presence of vesicular nuclei with large nucleoli, a high nuclear-cytoplasmic ratio, cell flocculation, pleomorphism, high mitotic rate, and necrosis suggest a malignant appearance of the tumors. The presence of three of these criteria supports malignancy, while fewer than three criteria suggest an atypical GCT [14].

When possible, surgical resection with clear margins is the standard treatment. An incomplete resection requires further surgical intervention due to the risk of local recurrence. For malignant forms, a margin of 2-3 cm is recommended due to the high risk of local recurrence (32-41% of cases) and metastasis (50-63% of cases) [15]. Patients undergoing chemotherapy and radiation therapy are few, as studies have shown that these treatments are not very effective. However, the role of radiation therapy remains controversial in the literature [16], while chemotherapy has not been proven to be effective [17].

Benign and atypical GCTs have a favorable course with no metastatic potential, while malignant GCTs have a poor prognosis with local recurrence and metastasis rates of 32% and 50%, respectively [14], and a 39% mortality rate at three years [18]. Factors such as older age, larger tumor size, local recurrence, metastasis, Ki67 >10%, and immunoreactivity for P53 are all poor prognostic indicators [18,19].

## Conclusions

GCTs are generally benign neoplasms, but their potential for malignant transformation, though rare, should not be overlooked, especially in cases of recurrence. This case highlights the importance of vigilant follow-up and early detection of malignant features in GCTs, as metastasis can occur even after initial benign presentations. Surgical resection with clear margins remains the cornerstone of treatment, but the prognosis for malignant GCTs is poor, with high recurrence rates and limited response to chemotherapy and radiation therapy. Multidisciplinary management and regular monitoring are essential for improving outcomes in such rare and aggressive cases.
